# Veratridine Induces Vasorelaxation in Mouse Cecocolic Mesenteric Arteries

**DOI:** 10.3390/toxins16120533

**Published:** 2024-12-10

**Authors:** Joohee Park, Christina Sahyoun, Jacinthe Frangieh, Léa Réthoré, Coralyne Proux, Linda Grimaud, Emilie Vessières, Jennifer Bourreau, César Mattei, Daniel Henrion, Céline Marionneau, Ziad Fajloun, Claire Legendre, Christian Legros

**Affiliations:** 1 Univ. Angers, INSERM, CNRS, MITOVASC, Equipe CarME, SFR ICAT, 49000 Angers, France; pakjoohee25@gmail.com (J.P.); christina.sahyoun1@gmail.com (C.S.); jacynthefrangieh@gmail.com (J.F.); lea.rethore@univ-angers.fr (L.R.); coralyne.proux@univ-angers.fr (C.P.); linda.grimaud@univ-angers.fr (L.G.); emilie.vessieres@univ-angers.fr (E.V.); jennifer.bourreau@inserm.fr (J.B.); cesar.mattei@univ-angers.fr (C.M.); daniel.henrion@univ-angers.fr (D.H.); claire.legendre@univ-angers.fr (C.L.); 2 Laboratory of Applied Biotechnology (LBA3B), Department of Cell Culture, Azm Center for Research in Biotechnology and Its Applications, EDST, Lebanese University, Tripoli 1300, Lebanon; ziad.fajloun@ul.edu.lb; 3 Nantes Université, CNRS, INSERM, l’Institut du thorax, 44000 Nantes, France; celine.marionneau@univ-nantes.fr; 4 Department of Biology, Faculty of Sciences 3, Campus Michel Slayman Ras Maska, Lebanese University, Tripoli 1352, Lebanon

**Keywords:** veratridine, voltage-gated Na^+^ channel, Na^+^/Ca^2+^ exchanger (NCX), mouse mesenteric arteries, myography, mesenteric and endothelial cell lines

## Abstract

The vegetal alkaloid toxin veratridine (VTD) is a selective voltage-gated Na^+^ (Na_V_) channel activator, widely used as a pharmacological tool in vascular physiology. We have previously shown that Na_V_ channels, expressed in arteries, contribute to vascular tone in mouse mesenteric arteries (MAs). Here, we aimed to better characterize the mechanisms of action of VTD using mouse cecocolic arteries (CAs), a model of resistance artery. Using wire myography, we found that VTD induced vasorelaxation in mouse CAs. This VTD-induced relaxation was insensitive to prazosin, an α1-adrenergic receptor antagonist, but abolished by atropine, a muscarinic receptor antagonist. Indeed, VTD–vasorelaxant effect was totally inhibited by the Na_V_ channel blocker tetrodotoxin (0.3 µM), the NO synthase inhibitor L-NNA (20 µM), and low extracellular Na^+^ concentration (14.9 mM) and was partially blocked by the NCX1 antagonist SEA0400 (45.4% at 1 µM). Thus, we assumed that the VTD-induced vasorelaxation in CAs was due to acetylcholine release by parasympathetic neurons, which induced NO synthase activation mediated by the NCX1-Ca^2+^ entry mode in endothelial cells (ECs). We demonstrated NCX1 expression in ECs by RT-qPCR and immunohisto- and western immunolabelling. VTD did not induce an increase in intracellular Ca^2+^ ([Ca^2+^]i), while SEA0400 partially blocked acetylcholine-triggered [Ca^2+^]i elevations in Mile Sven 1 ECs. Altogether, these results illustrate that VTD activates Na_V_ channels in parasympathetic neurons and then vasorelaxation in resistance arteries, which could explain arterial hypotension after VTD intoxication.

## 1. Introduction

Veratridine (VTD) is a steroidal alkaloid from the seeds of the *Veratrum* species, representing one the oldest known selective ligands of voltage-gated Na^+^ (Na_V_) channels [1,2,3]. Through binding to Na_V_ channel site 2 within the pore, VTD increases Na^+^ current duration by inhibiting current inactivation and, thereby, inducing membrane depolarization and, consecutively, extracellular Ca^2+^ entry [3,4,5]. VTD is responsive to mild intoxication in humans, leading to gastro-intestinal, neurological, cardiac, and vascular symptoms that can be alleviated with the parasympatholytic drug atropine [6,7]. The arterial hypotension caused by VTD has been earlier attributed to the diminution of peripheral vascular resistance, a primary determinant of arterial blood pressure [1,8,9]. Like many plant alkaloids, VTD was used in the past to treat arterial hypertension, but the mechanism by which it decreases arterial blood pressure is still not completely understood [8,10].

Indeed, VTD has been used to investigate the contribution of Na_V_ channels in isolated arteries, in which they are expressed in peripheral nerves, in smooth muscle cells (SMCs) and endothelial cells (ECs) [11,12,13,14]. In conduction arteries, such as aorta and femoral arteries, VTD triggers vasoconstriction mediated by Ca^2+^ entry in SMCs through the Na^+^/Ca^2+^ exchanger (NCX) [12,13,15,16,17]. As for peripheral small arteries, which contribute to peripheral vascular resistance, VTD induces the vasoconstriction of rat mesenteric arteries and human uterine arterioles, also mediated by the NCX Ca^2+^ entry mode in SMCs [12,17]. In contrast, VTD induces the vasodilation of mouse cremaster and pig retinal arterioles, underlying endothelium contribution [18,19]. However, the mechanisms behind VTD-induced vasorelaxation have not been clearly defined on isolated arteries.

**Figure 1:**
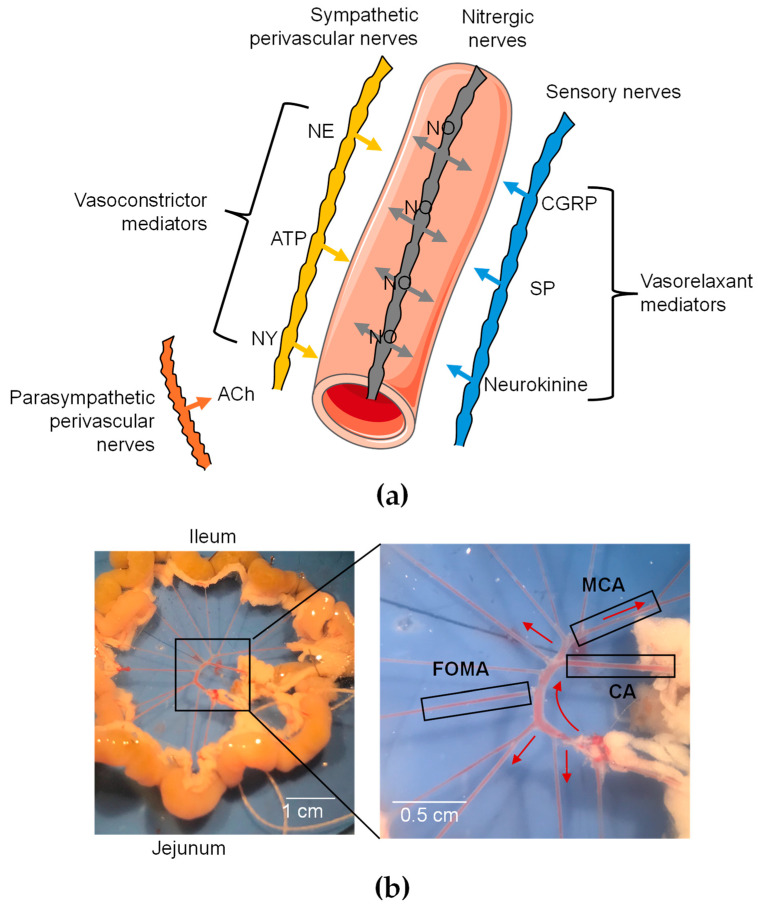
Functional organization of perivascular innervation of mesenteric arteries. (**a**) Schematic representation of periarterial innervation. Neurotransmitters are released from varicosities and diffuse to receptors in SMCs and ECs. CGRP = calcitonin gene-related peptide; NE = norepinephrine; NY = neuropeptide Y; SP = substance P. Galanin is not shown since it has not been detected in nerve fibers of mesenteric arteries. (**b**) Image of mesenteric arterial bed in mice. Three different branches were used: cecocolic artery (CA; diameter = ~200 µm), middle colic artery (MCA; diameter = ~150 µm), and first-order mesenteric artery (FOMA; diameter = ~150 µm). Red arrow illustrates blood flow.

Peripheral vascular resistance is tightly controlled by vascular innervation releasing different neurotransmitters by sensory motors (CGRP, ATP, substance P, neurokinin A, and anadamide) and sympathetic nerves (norepinephrine, ATP, neuropeptide Y, and galanin) (Figure 1a) [20,21]. While norepinephrine (NE), ATP, and neuropeptide Y, released by sympathetic nerves, are vasoconstrictor mediators, CGRP, substance P, and neurokinin, released by sensory neurons, are vasodilators [20,21]. However, the parasympathetic control of resistance arteries is controversial. While cholinergic nerves have been observed in mesenteric arteries, their vasomotor functions are not associated with vascular tone regulation [22]. In isolated arteries, VTD can thus activate Na_V_ channels either in neuronal networks, in ECs, or even in SMCs. Consequently, the resulting contractile responses could be tricky to interpret.

Hence, the present study aimed to better understand the mode of action of VTD on isolated mouse mesenteric arteries (MAs) by using wire myography. We found that VTD induced relaxation in cecocolic arteries (CAs) and middle colic arteries (MCAs), two distinct branches of MAs (Figure 1b). Using a pharmacological approach, we explored the contribution of the vascular innervation in VTD-induced vasorelaxation. Notably, we found that VTD-induced vasorelaxation involves the activation of endothelial NO synthase (eNOS), mediated by the increase in intracellular Ca^2+^ ([Ca^2+^]i) through the NCX Ca^2+^ entry mode in ECs.

## 2. Results

### 2.1. VTD Induces Vasorelaxation in CAs and MCAs

**Figure 2:**
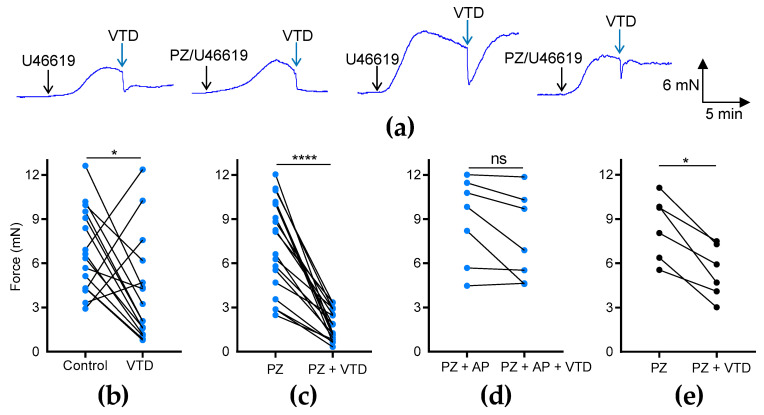
Effects of VTD on CAs and MCAs. Wall tension generated by CAs and MCAs was measured with and without PZ (1 µM). (**a**) Examples of myographic traces showing effects of VTD (30 µM) on CAs. (**b**–**e**) Graphs illustrating connected scatter plots of individual values of wall tension levels (force, in mN) before and after VTD application for each CA (**b**–**d**) and MCA (**e**). Control: wall tension with U46619 alone; PZ: wall tension with U46619 with PZ; PZ + AP: wall tension with U46619, PZ, and atropine (AP, 100 nM). CAs were isolated from male (*n* = 9) and female (*n* = 7) mice (**b**,**c**). Only male mice were used in (**d**) (*n* = 7) and (**e**) (*n* = 6). Significance was evaluated using paired *t* test: *: *p* < 0.05; ****: *p* < 0.0001; ns: non-significant; *p* = 0.0602.

To investigate the effects of VTD on the vascular tone of MAs, we used two different arterial branches, CAs and MCAs (Figure 1b), which were stimulated beforehand with an analog of thromboxane A2, U46619, to reach 50% of the maximal contraction (Figure 1b). In the CAs, we observed that VTD triggered two types of responses: a sustained or transient vasorelaxant response that was insensitive to prazosin (PZ) and an α1-adrenergic receptor antagonist (Figure 2a). The analysis of the myographic data indicated that VTD (30 µM) induced a significant decrease in the wall tension with or without 1 µM PZ (*p* < 0.05; Figure 2b,c). However, the relaxation was significantly higher in the presence of PZ (77.1 ± 3.8%; *n* = 14) than in its absence (50.7 ± 13.5%; *n* = 21; *p* < 0.05; Mann–Whitney test).

The addition of the muscarinic receptor antagonist atropine (AP, 100 nM) totally suppressed the VTD-induced relaxation of the CAs from males (*p* = 0.0602; Figure 2d). In the MCAs, we also found that VTD triggered vasorelaxation in the presence of PZ (*p* < 0.05; Figure 2e). Therefore, these data show that VTD induced vasorelaxation in the CAs and MCAs. In addition, since the addition of PZ increased the VTD–vasorelaxant effect, while this effect was inhibited by AP, we concluded that VTD induced the release of both NE and ACh from perivascular nerves in the CAs.

**Figure 3:**
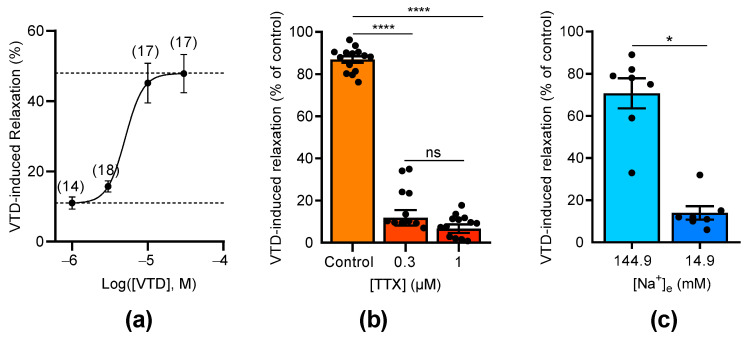
Concentration-dependent effects of VTD and TTX on CAs. (**a**) Concentration–relaxation relationship of VTD effect on CAs analyzed using Langmuir equation (EC_50_ = 5 µM; maximum effect = 47.9%). Numbers in brackets indicate the numbers of animal used. (**b**) Scatter plots illustrating effect of TTX (0.3 and 1 µM) on VTD-induced relaxation (30 µM). (**c**) Scatter plots showing effect of low extracellular Na^+^ concentration ([Na^+^]e = 14.9 mM) on VTD-induced relaxation (30 µM). All experiments were performed with PZ (1 µM). CAs were isolated from male (*n* = 10) and female (*n* = 10) mice (**a**,**b**). Only male mice were used in (**c**) (*n* = 7). Values are means ± SEM. Significance was analyzed with one-way ANOVA test followed by Dunn’s test (**b**) for multiple comparisons (ns: non-significant; ****: *p* < 0.0001) and paired *t* test (**c**) (*: *p* < 0.05).

The concentration–relaxation relationship of VTD showed a relaxation of the CAs with a maximum effect of 47.9% at 30 µM and a half-maximal effective concentration (EC_50_) of 5 µM (Figure 3a). As expected, the VTD-induced relaxation responses were suppressed by the Na_V_ channel inhibitor tetrodotoxin (TTX, Figure 3b). Indeed, at 0.3 µM of TTX, the VTD-induced relaxation responses were totally inhibited, and no more inhibition was obtained with 1 µM, indicating that VTD binds to TTX-sensitive Na_V_ channels in CAs (Figure 3b). Since VTD induces permanent extracellular Na^+^ entry, we also examined the effects of lowering the extracellular Na^+^ concentration to 14.9 mM instead of 144.9 mM, which suppressed the Na^+^ concentration gradient. As expected, the VTD-induced vasorelaxation effect was significantly decreased by 5.1 folds (*p* < 0.05; Figure 3c).

### 2.2. VTD-Induced Vasorelaxation in CAs Is Mediated by the NO-Pathway

**Figure 4:**
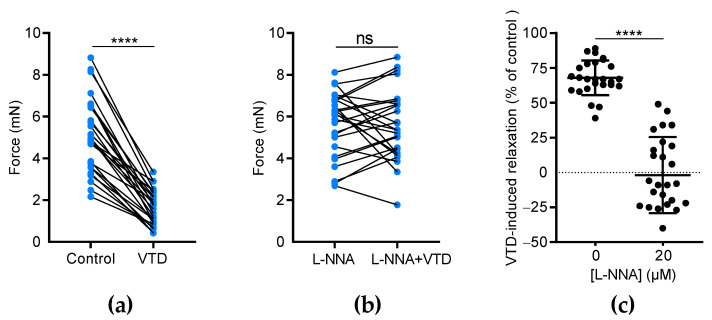
The effects of L-NNA on VTD-induced vasorelaxation in CAs. (**a**,**b**) Connected scatter plots showing the contractile force (in mN) generated by isolated CAs before and after the application of VTD (30 µM) in the absence of L-NNA (**a**) and in the presence of 20 µM L-NNA (**b**). (**c**) Scatter plots illustrating the reduction in VTD-induced relaxation (in %) by L-NNA. All experiments were carried out on pre-contracted CAs with U46619 in the presence of PZ (1 µM). The CAs were isolated from male (*n* = 16) and female (*n* = 10) mice. The significance was analyzed by a parametric paired *t* test (****: *p* < 0.0001; ns: non-significant; *p* = 0.8193).

We assumed that VTD-induced vasorelaxation in CAs is likely due to ACh release by parasympathetic nerves [22]. Since ACh stimulates the production of the vasorelaxant endothelial factor NO, we investigated the effects of N(ω)-nitro-L-arginine (L-NNA), a NO synthase inhibitor, on VTD-induced vasorelaxation. In this set of experiments, each U46619-contracted artery was stimulated by VTD in the presence of PZ and, after washing, the experiment was repeated in the presence of L-NNA (20 µM, Figure 4). As described above, at 30 µM, VTD induced vasorelaxation in CAs (32.1 ± 2.4%; *n* = 26; *p* < 0.0001) while, in the presence of L-NNA, VTD had no longer had any effect on the contraction of CAs (*p* = 0.8193; Figure 4a,b). Since L-NNA totally inhibited the VTD-induced vasorelaxant responses (*p* < 0.0001; Figure 4c), we concluded that VTD induced ACh release by parasympathetic nerves which, in turn, activated NO production in the endothelium, leading to vasorelaxation.

### 2.3. NCX Inhibition Partially Abolished VTD-Induced Vasorelaxation in CAs

**Figure 5:**
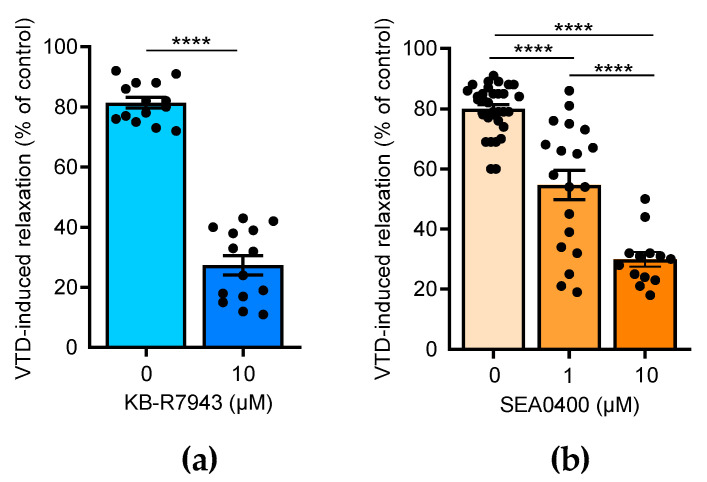
Effects of NCX antagonists on VTD-induced vasorelaxation in CAs. Scatter plots illustrating reduction in VTD-induced vasorelaxant responses of CAs with KB-R7943 (10 µM, NCX blocker) (**a**) and with SEA0400 (1 and 10 µM, NCX1 blocker) (**b**). CAs were pre-contracted with U46619 in presence of PZ (1 µM). CAs were isolated from males (*n* = 7) and females (*n* = 7) (**b**). Values are means ± SEM. Significance was analyzed with paired *t* test (**a**) and one-way ANOVA, followed by Tukey multiple comparison test (**b**) (****: *p* < 0.0001).

Few studies have reported that NCX is also expressed in the endothelium and contributes to the activation of NO production in ECs [23,24,25]. Thus, we assayed the effects of 2-[2-[4-(4-nitrobenzyloxy)phenyl]ethyl]isothiourea methane sulfonate (KB-R7943), which blocks NCX1-3, and 2-[4-[(2,5-difluorophenyl)methoxy]-phenoxy]-5-ethoxyaniline (SEA0400), a selective blocker of NCX1 [26]. In the presence of KB-R7943 (10 µM), the VTD-induced vasorelaxant responses were strongly decreased by 3.0 folds (*p* < 0.0001; Figure 5a). SEA0400 also significantly reduced the VTD-induced vasorelaxation in a concentration-dependent manner by 1.5 and 2.7 folds, at 1 and 10 µM, respectively (*p* < 0.0001; Figure 5b). These results indicate that NCX likely contributes to the activation of endothelial NO synthase (eNOS) in the endothelium in response to VTD-induced ACh release.

**Figure 6:**
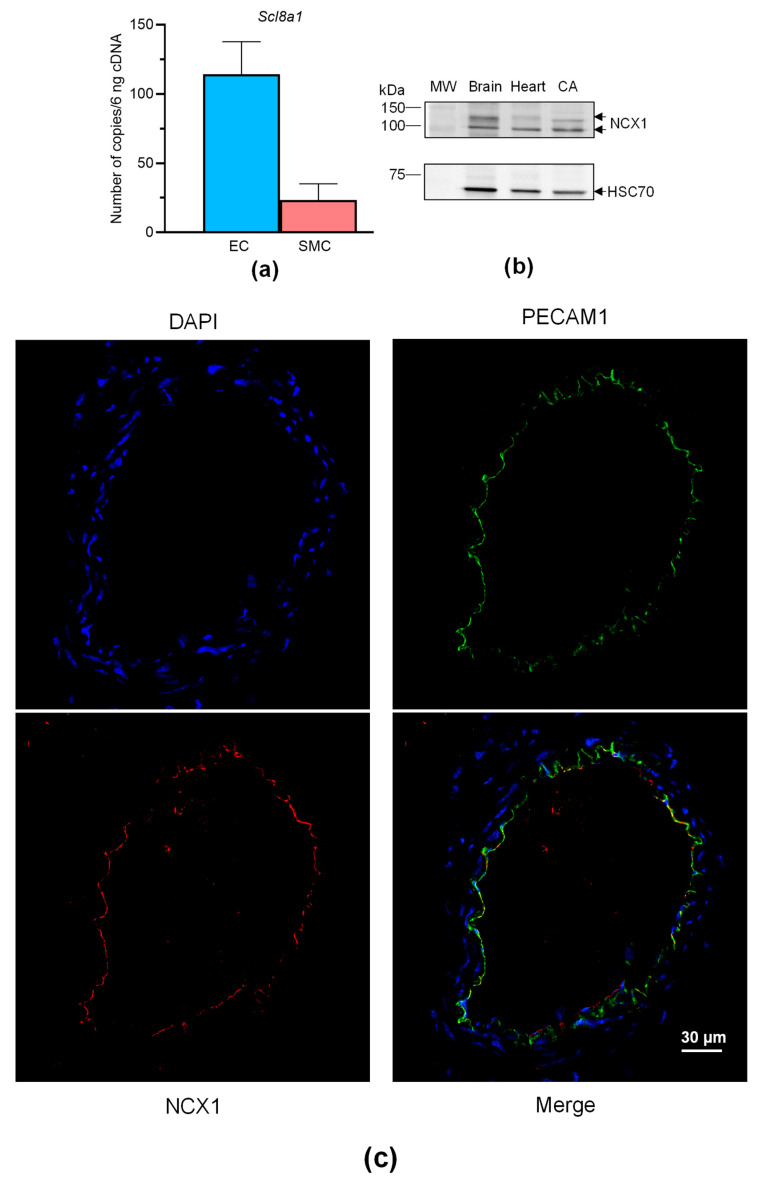
NCX expression in CAs. (**a**) Histograms showing the mRNA expression levels of NCX determined by absolute RT-qPCR in CAs collected from male and female mice. (**b**) NCX1 immunoblotting in the CAs. NCX1 expression was evaluated in CAs by Western blotting with an anti-NCX1 antibody. HSC70 was used as a loading control and mouse brains and hearts were used as positive control samples. The experiments were carried out with 10 µg of proteins. Due to the limited size of the CAs, segments of three mice were pooled. (**c**) NCX1 immunolocalization in CAs. NCX1 were immunodetected in CAs by an anti-NCX1 antibody (red). Endothelium was immunolabeled by an anti-PECAM1 antibody (green). The nucleus was labeled by DAPI (blue). Values are the means ± SEM of three independent experiments. MW: molecular weight.

Overall, these findings show that VTD binds to Na_V_ channels in perivascular nerves, leading to NO-dependent vasorelaxation induced by ACh, involving the contribution of NCX. Like Na_V_ channels, NCX is expressed in neurons, SMCs, and ECs [27], and both can be functionally coupled in these cells [11,17,28]. We have previously shown that Na_V_ channels are expressed in SMCs and ECs of mouse CAs [29], but NCX expression has not yet been described in this artery. Hence, we first characterized the expression of NCX in CAs, ECs and SMCs isolated from mouse mesenteric arterial beds. The absolute RT-qPCR data show that only *Slc8a1*, encoding NCX1, was expressed in ECs and SMCs isolated from MAs (Figure 6a). *Slc8a2* (encoding NCX2) and *Slc8a3* (encoding NCX3) were not detectable. We then confirmed the expression of NCX1 by Western blotting and immunohistochemistry. The anti-NCX1 antibodies allowed the immunostaining of two bands with molecular weights corresponding to NCX1 with protein extracts from CAs (Figure 6b). In addition, strong immunolabeling was observed in CAs, exclusively in ECs and in co-localizing with the platelet EC adhesion molecule (PECAM1), an endothelium marker (Figure 6c).

### 2.4. VTD Has No Effects on Intracellular Ca^2+^ in ECs

**Figure 7:**
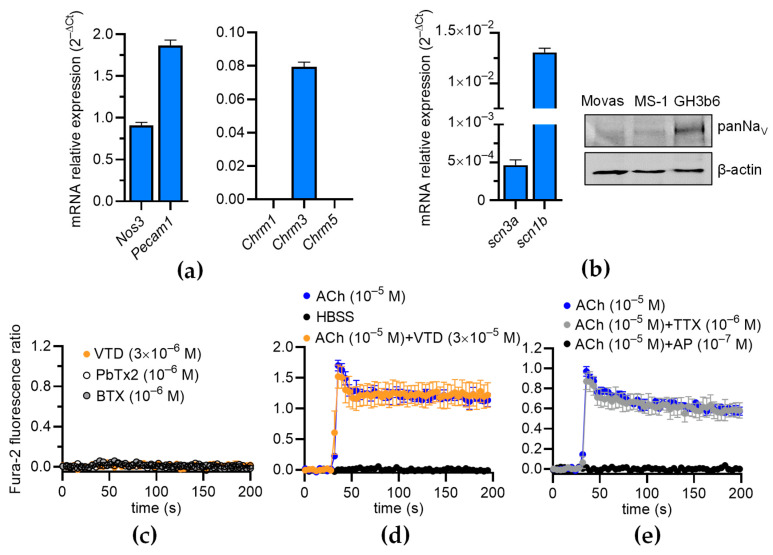
Effects of VTD on intracellular Ca^2+^ concentration in MS1 ECs. (**a**,**b**) RT-qPCR data, illustrated as RNA relative level (2^−ΔCT^), showing expression of *nos3*, *pecam1*, *chrm1-3*, *scn3a*, and *scn1b*. Undetectable genes are not represented (*scn1a*, *scn2a*, *scn4a*, *scn5a*, *scn8-11a*, *scn1-2b*, and *scn4b*) (**b**) Western blot (right panel) illustrating Na_V_ channel expression in MS1 ECs. Western blot was performed with 30 µg of proteins. PanNa_V_ and β-actin antibodies were used. GH3b6 cells were used as positive control. (**c**,**d**) Example of kinetic traces of Fura-2 fluorescence–emission ratio obtained before and after injection, at 30 s, of VTD, brevetoxin 2 (PbTx2), batrachotoxin (BTX) (**c**), and ACh (**d**,**e**). Effects of co-injection of ACh and VTD or TTX or AP are shown (**d**,**e**). Hank’s Balanced Salt Solution (HBSS) was used as negative control. Data are the mean ± SEM of three independent experiments (*n* = 3).

Since Na_V_ channels are expressed in the ECs of FOMAs and CAs [29], and a functional coupling between these channels and NCX has been evidenced in human umbilical ECs (HUVECs) [11], we investigated whether VTD could induced a Ca^2+^ response in mouse ECs, which can induce the activation of eNOS [23,24,25]. The RT-qPCR data show that the Mile Sven 1 pancreatic ECs (MS1 ECs) expressed *Nos3*, encoding eNOS, and the EC markers *Pecam1* and *Chrm3*, encoding the muscarinic receptor subtype 3 (M3-mAChR), showing that they constitute a convenient model for our study (Figure 7a). In addition, we found that the MS1 ECs expressed *scn3a* and *scn1b*, encoding the Na_V_ channel subunits Na_V_1.3 and Na_V_β1 (Figure 7b). The following genes were undetectable: *scn1a*, *scn2a*, *scn4a*, *scn5a*, *scn8-11a*, and *scn2-4b.* Na_V_ channel expression was also evidenced by Western blotting (Figure 7b). At 30 µM, VTD did not modify [Ca^2+^]i in the MS1 ECs, despite the endogenous expression of Na_V_ channels, nor Na_V_ channel activators such as batrachotoxin (BTX) and brevetoxin 2 (PbTx2) (Figure 7c). In addition, while ACh induced a clear [Ca^2+^]i elevation, this response was not affected by the co-injection of VTD, nor by TTX (Figure 7d,e). As expected, the ACh-induced Ca^2+^ response was totally inhibited by AP (Figure 7e). These observations are thus consistent with our assessment that the VTD-induced relaxation of CAs is due to Na_V_ channel activation in cholinergic perivascular nerves, leading to ACh release, and not to a direct effect of VTD in ECs.

### 2.5. NCX1 Contributes to ACh-Induced Ca^2+^ Responses in ECs

**Figure 8:**
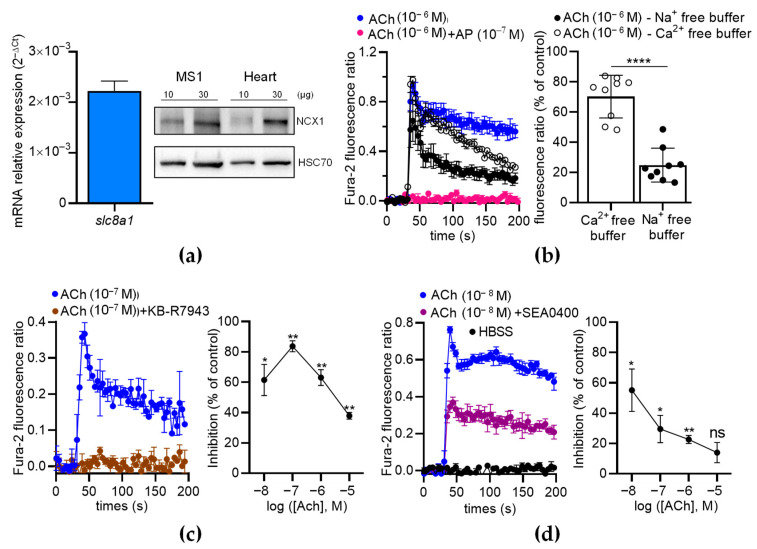
The effects of NCX antagonists on the ACh-induced Ca^2+^ response in MS1 ECs. (**a**) RT-qPCR data (**left panel**) illustrated as the RNA relative level (2^−ΔCT^), showing the expression of *slc8a1*, encoding NCX1. The undetectable genes (*Slc8a2* and *Slc8a3*) are not illustrated. The Western blot (**right panel**) shows the immunodetection of NCX1 in the MS1 ECs. HSC70 antibodies were used as loading controls. Protein extracts from mouse hearts served as positive controls. (**b**) An example of kinetic traces of the Fura-2 fluorescence–emission ratio, illustrating the effects of ACh (1 µM) co-injected or not co-injected with AP (100 nM), in free Na^+^ and Ca^2+^ buffers. The histograms illustrate the normalized emission ratio of Fura-2 measured after Ach injection (1 µM) in Ca^2+^-free buffer and in Na^+^-free buffer. These data represent the area under the curve (AUC) calculated from the kinetic traces and after normalization by ACh-induced responses at 1 µM in Hank’s Balanced Salt Solution (HBSS), used as the control. (**c**,**d**) The effects of NCX antagonists on the ACh-induced Ca^2+^ response in MS1 ECs. The left panels show examples of kinetic traces of the Fura-2 fluorescence–emission ratio before and after the injection, at 30 s, of ACh, which led to the highest inhibition induced by the two NCX antagonists KB-R7943 (10 µM) (**c**) and SEA0400 (10 µM) (**d**). The graphs on the right panels illustrate the inhibition (% of control) induced by KB-R7943 (**c**) and SEA0400 (**d**), as a function of the ACh concentration. HBSS was used as a negative control (**d**). Data are the mean ± SEM of three independent experiments (*n* = 3). Statistical significances were determined using the Mann–Whitney test (**b**) and Wilcoxon test (**c**,**d**) (* *p* < 0.05; ** *p* < 0.01; **** *p* < 0.0001; ns: non-significant).

To further analyze the contribution of NCX in ECs, we first characterized the expression of NCX in MS1 ECs. We found that the MS1 ECs endogenously expressed *Slc8a1* (Figure 8a, left panel), while *Slc8a2* and *Slc8a3* were undetectable by RT-qPCR. The expression of NCX1 was confirmed at the protein level by Western blotting (Figure 8a, right panel). As expected, the ACh-induced Ca^2+^ increase was completely inhibited by AP (100 nM), indicating that ACh-induced Ca^2+^ responses are mediated by muscarinic receptors (Figure 8b, left panel). In the absence of extracellular Ca^2+^, the Ach-induced Ca^2+^ responses exhibited a peak with the same amplitude as the control, followed by a slow decrease in the signal over time (Figure 8b, left panel). However, in the absence of extracellular Na^+^, the ACh-induced Ca^2+^ responses were considerably reduced (Figure 8b, left panel). Indeed, the ACh-induced Ca^2+^ responses decreased to 70.23 ± 4.72% (*n* = 9; *p* < 0.01; Wilcoxon test) in the absence of extracellular Ca^2+^ and dropped to 24.83 ± 3.75% (*n* = 9; *p* < 0.01; Wilcoxon test) in the absence of extracellular Na^+^ (Figure 8b, right panel). The ACh-induced Ca^2+^ responses were significantly lower in the absence of extracellular Na^+^ than in the absence of extracellular Ca^2+^ (*p* < 0.0001) (Figure 8b, right panel). Thus, the ACh-induced Ca^2+^ responses in the ECs depended on extracellular Ca^2+^ and extracellular Na^+^, in agreement with the contribution of NCX via the Ca^2+^ entry mode [30].

Then, we used two inhibitors of the Ca^2+^ entry mode of NCX: KB-R7943 and SEA0400. The ACh-induced Ca^2+^ responses were much lower when ACh was co-injected with KB-R7943 (10 µM) or SEA0400 (10 µM) (Figure 8c,d). With KB-R7943, the inhibition rate depended on the ACh concentrations (Figure 8c, right panel). Notably, the highest inhibition rate occurred at 10^−7^ M of ACh (87.7% of inhibition based on the area under the curve determination (AUC)) and progressively decreased with increasing ACh concentrations (Figure 8c, right panel). The inhibitory effects of SEA0400 on the Ca^2+^ responses were the highest at 10^−8^ M of ACh and decreased with increasing ACh concentrations (Figure 8d, right panel). Similar results were observed with a third inhibitor of the Ca^2+^ entry mode of NCX, i.e., 2-[[4-[(4-Nitrophenyl)methoxy]phenyl]methyl]-4-thiazolidinecarboxylic acid ethyl ester (SN-6) at 10 µM (Figure S1).

Altogether, our data show that NCX1 contributes to [Ca^2+^]i elevation induced by ACh in MS1 ECs.

### 2.6. Thapsigargin and Chelerythrine Reduce ACh-Induced Ca^2+^ Responses in ECs

**Figure 9:**
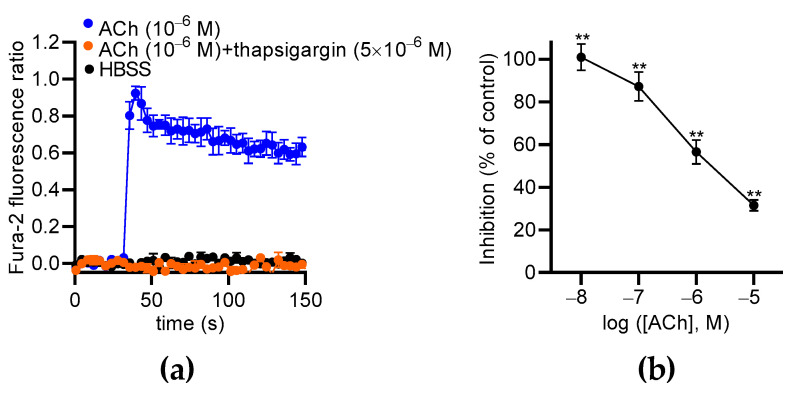
The effects of thapsigargin and chelerythrine on ACh-induced Ca^2+^ responses in MS1 ECs. (**a**) The effects of thapsigargin (5 µM) on Ca^2+^ responses induced by 1 µM of ACh. Hank’s Balanced Salt Solution (HBSS) was used as a negative control. (**b**) The curves represent the inhibitory effects of chelerythrine (10 µM) on the Ca^2+^ responses induced by 0.01, 0.1, 1, and 10 µM of ACh in the MS1 ECs. The analyses were based on the area under the curve determined from the kinetic traces. The data were normalized by the Ca^2+^ response induced by ACh at 10 µM. The mean values of the inhibitory effects were compared to 0% using a one-sample *t* test (** *p* < 0.01). Values are means ± SEM (*n* = 3).

Our data show that extracellular Ca^2+^ partially contributes to ACh-induced Ca^2+^ responses in MS1 ECs, suggesting the involvement of Ca^2+^ store mobilization, as already described in other ECs [31,32,33]. Thus, we used thapsigargin to deplete the endoplasmic reticulum (ER) Ca^2+^ store in the MS1 ECs and measure the effects of ACh. We found that thapsigargin (5 µM) totally suppressed ACh-induced Ca^2+^ responses in the MS1 ECs (Figure 9a). Thus, the cytosolic elevation of Ca^2+^ was mainly mediated by store-operated channels, such as inositol 1,4,5 triphosphate receptors. Because the Ca^2+^ signaling pathway activated by ACh leads to PKC activation, which is known to increase NCX1 activity by phosphorylation [34,35], we tested the effects of its inhibition with chelerythrine, a pan-PKC inhibitor [36]. ACh-induced Ca^2+^ responses were measured with the same protocol used for testing NCX inhibitors. We observed that chelerythrine suppressed Ca^2+^ responses induced by 10^−8^ M of ACh (100.0% inhibition; *p* < 0.01). The inhibitory effects of chelerythrine progressively decreased when the ACh concentration was increased to reach 31.6% (*p* < 0.01) of the inhibition with 10^−5^ M of ACh (Figure 9b).

In conclusion, ACh induces Ca^2+^ release from ERs and then the activation of PKC that likely stimulates NCX1 in the Ca^2+^ entry mode.

## 3. Discussion

In the present study, we show that VTD induces vasorelaxation in CAs and MCAs, implicating the activation of Na_V_ channels. Since VTD-induced vasorelaxation in the CAs was higher in the presence of PZ and totally inhibited by the muscarinic receptor antagonist AP, we assumed that this contractility response was the consequence of NE and ACh releases from sympathetic and parasympathetic perivascular nerves, respectively. The VTD-induced vasorelaxation in the CAs was inhibited by AP and L-NNA, suggesting that this response was mediated by NO production in ECs. In addition, we found that VTD-induced vasorelaxation is mediated by NCX. Using MS1 ECs, we showed that VTD had no effect on [Ca^2+^]i in the ECs, even though they expressed Na_V_ channels at both transcriptional and protein levels. This reinforces the idea that VTD activates Na_V_ channels in cholinergic perivascular nerves, leading to ACh release and endothelium-dependent vasorelaxation. Finally, we evidenced that, in MS1 ECs, ACh induces cytosolic Ca^2+^ elevation involving NCX1 in the Ca^2+^ entry mode.

We first aimed to study the role of Na_V_ channels, endogenously expressed in ECs and vascular smooth muscle cells (VSMCs), in the vasomotor regulation of small arteries. Indeed, VTD has been extensively used to study the implication of vascular Na_V_ channels in the contraction of isolated arteries [12,13,15,18,37]. However, the ⍺-1 adrenoceptor antagonist PZ strongly reduces VTD-induced contraction in the first-order MAs of rats [12] and suppresses the vasoconstriction of rat aortas [13] and human uterine arteries [37]. Our present findings, showing that VTD-induced vasorelaxation in MCAs is suppressed by AP, are in agreement with these reports. Thus, VTD activates the Na_V_ channels of perivascular nerves, and its vascular effects arise from the release of NE or ACh, depending on the artery. These observations raise a question concerning the contribution of Na_V_ channels in both arterial SMCs and ECs in vascular motricity. We have previously shown that TTX induces vasorelaxation in mouse CAs in the presence of VTD, PZ, and AP, indicating that arterial Na_V_ channels can be activated during arterial constriction [29]. In this context, the vascular effects of VTD must be carefully considered, and the role of Na_V_ channels endogenously expressed in ECs and VSMCs remains unclear under physiological conditions.

Thus, the contractile effect of VTD on MAs depends on the branch type, reflecting differences in vasomotor innervation. Indeed, VTD induces contraction in FOMAs from rats [12] and mice (Figure S2), while it induces vasorelaxation in CAs and MCAs. These arteries are known to be densely innervated, and, notably, it is well established that FOMAs and other branches of cranial mesenteric arteries are innervated by both adrenergic- and calcitonin gene-related peptide (CGRP) nerves that are vasoconstrictor and vasodilator, respectively [22,38,39,40]. None or few cholinergic nerves have been detected in FOMAs, and they are not associated with vasomotor regulation [22]. Thus, our data are consistent with these statements, as VTD-induced vasoconstriction in FOMAs is abolished by the ⍺-1 adrenoceptor antagonist (Figure S2). However, the innervation of CAs has not been described in detail, but the fact that the VTD-induced vasorelaxation was abolished by the muscarinic receptor antagonist strongly suggests that this branch contains vasomotor cholinergic nerves. These differences in the vasomotor innervation between the FOMAs and CAs likely reflect differences in jejunum–ileum and cecum vascularization to allow the fine control of blood flow throughout the mesenteric bed. Further experiments would help to confirm the distribution and function of cholinergic nerves in CAs.

Here, we assume that VTD induced ACh release from parasympathetic nerves and subsequently endothelial NO production, which triggered vasorelaxation. Indeed, we observed that L-NNA suppressed VTD-induced vasorelaxation in CAs. Since eNOS activity is regulated by Ca^2+^-dependent and phosphatidylinositol-3 kinase/Akt pathways, we can postulate that VTD indirectly evokes [Ca^2+^]i elevation in ECs [41]. In addition, regarding that ACh induced [Ca^2+^]i elevation mediated by G_q/11_-coupled muscarinic receptors, we assume that this pathway was involved in the eNOS activation observed in the VTD-treated CAs. Moreover, the NCX antagonists KB-R7943 and SEA0400 significantly reduced VTD-induced vasorelaxation in the CAs, suggesting that endothelial NCX contributes to [Ca^2+^]i elevation and, subsequently, to NO production, as previously described [23,24,25]. This assessment is supported by our observation of the expression of NCX1 in the endothelium of the CAs and in the dissociated ECs. NCX is also expressed in neurons [27], but since the mAChR antagonist AP abolishes VTD-induced vasorelaxation in CAs, we could exclude its contribution.

VTD did not affect [Ca^2+^]i concentration in the MS1 ECs. Thereby, there was not functional coupling between Na_V_ channels and Ca^2+^ homeostasis in these cells, as has been described in HUVECs [11]. This suggests that Na_V_ channels are not involved in the regulation of NO production in the endothelium. Thus, we could exclude the implication of endothelial Na_V_ channels in the VTD-induced relaxation observed in the CAs, which was more likely due to ACh release and, in turn, store-operated Ca^2+^ release, leading to eNOS activation [42]. By monitoring [Ca^2+^]i, we found that primary ECs from the MAs and MS1 ECs exhibited similar responses to ACh, with a peak and a sustained component, as previously described [43]. The ACh-induced Ca^2+^ responses in both cell models were initiated by Ca^2+^ release from intracellular stores, as they were inhibited by incubation with thapsigargin. Moreover, ACh-induced [Ca^2+^]i elevation was significantly reduced in the Ca^2+^-free or Na^+^-free media, in agreement with previous studies [24].

Our data evidenced that only *Slc8a1* transcripts were expressed in dissociated ECs from the MAs and MS1 ECs, indicating that only NCX1 was expressed in the endothelium. Thus, the reduction in ACh-induced Ca^2+^ elevation by NCX inhibitors in the MS1 ECs revealed the contribution of the Ca^2+^ exchange entry mode of NCX1 in the Ca^2+^ signaling. This mechanism could explain why NCX and eNOS inhibitors also reduced VTD-induced vasorelaxation in the CAs. Since L-NNA totally suppressed the VTD-induced vasorelaxation in the CAs, we did not explore the implication of NCX1 in the endothelium-derived hyperpolarizing factor (EDHF) pathway, although this pathway has been shown to contribute to vasorelaxation induced by ACh in rat MAs [23]. The Ca^2+^ entry mode of NCX1 in ECs has also been proven in other EC models, such as ECs from MAs, HUVECs, and cardiac microvessels [23,44,45]. Our findings bring novel evidence of the Ca^2+^ entry mode of NCX1 in the endothelium which must be considered a significant actor in Ca^2+^-dependent vasodilation.

We showed that the MS1 ECs expressed only the muscarinic M3-mAChR, and its activation by ACh induced Ca^2+^ release from the ERs and extracellular Ca^2+^ entry through NCX1. Indeed, M3-mAChR is coupled to G_q/11_ proteins that can activate phospholipase Cβ and the subsequent release of inositol 1,4,5-triphosphate (IP3) from the plasma membrane [46]. The release of IP3 in the cytoplasm triggers Ca^2+^ release from ERs, which constitutes the first step leading to [Ca^2+^]i elevation. NCX1 could participate in [Ca^2+^]i elevation in a second phase, as already described [23]. In this context, the activation of the Ca^2+^ entry mode of NCX1 is more complex to understand. TrpV4 and TrpC channels have also been shown to contribute to [Ca^2+^]i elevation induced by ACh and bradykinine in ECs [47,48], and both could lead to a [Na^+^]i increase that could in turn activate the Ca^2+^ entry mode of NCX1 [23]. Here, we can definitively exclude the involvement of Na_V_ channels in [Na^+^]i increase in MS1 ECs. Interestingly, our data showed that the activation of PKC, which certainly occurred after Ca^2+^ release from the ERs, could contribute to the activation of the Ca^2+^ entry mode of NCX1. Further experiments using RNAi or shRNA to inhibit the expression of PKC in ECs will be needed to confirm this assumption.

## 4. Conclusions

Our data demonstrate that the vasomotor responses mediated by VTD are due to the activation of Na_V_ channels expressed in perivascular nerves in mouse CAs. Indeed, VTD induces vasorelaxant response in CAs, which is suppressed by AP, revealing ACh release by cholinergic perivascular nerves. ACh release exclusively causes intracellular Ca^2+^ elevation, mediated by NCX1 in the endothelium, which activates eNOS. Finally, we propose that the Ca^2+^ entry mode of NCX1 could be activated by PKC. In addition, our data bring novel evidence that arterial hypotension observed during VTD poisoning could be partially attributed to a diminution of peripheral vascular resistance.

## 5. Materials and Methods

### 5.1. Chemicals and Reagents

VTD was obtained from Santa Cruz Biotechnology (Dallas, Texas, USA). TTX was obtained from Latoxan (Valence, France). U46619 was obtained from Cayman Chemical (Ann Arbor, MI, USA). Fura-2-AM and Optical Cutting Temperature (OCT) compounds were obtained from Thermo Fisher Scientific (Waltham, MA, USA). All other chemicals including ACh, phenylephrine (Phe), PZ, AP, L-NNA, KB-R7943, SEA0400, SN-6, thapsigargin, chelerythrine, and pluronic^®^-F127 acid were obtained from Sigma-Aldrich Merck (Saint-Louis, MO, USA).

### 5.2. Ethical Statement, Animal Care, and Conditioning

All animal procedures were realized in compliance with the European Community council directive 2010/63/EU for the care and use of laboratory animals and were approved by our local ethical committees (N° A49007002, Animal facilities Unit of the Hospital University of Angers (SCAHU)). All experiments using mice were conducted in accordance with the NC3R’s ARRIVE guidelines. Male and female C57BL/6j mice were purchased from Janvier Labs (Le Genest-Saint-Isle, France) and used at the age of 5–6 months. The mice were euthanized by CO_2_ inhalation in an automated chamber (Tem Sega, Pessac, France).

### 5.3. Wire Myography

The wire myograph technique was used to measure the contractility of the mice MAs, as already described, using a Mulvany–Halpern-type wire myograph (Danish MyoTechnology, DMT, Aarhus, Denmark) [29,49]. Two different branches of MAs, CAs and MCAs, were collected (Figure 1b) in cold physiological salt solution (PSS, in mM: NaCl, 130; KCl, 3.7; MgSO_4_(7H_2_O), 1.2; NaHCO_3_, 14.9; CaCl_2_(2H_2_O), 1.6; HEPES, 5; KH_2_PO_4_, 1.2; D-glucose, 11) at a pH of 7.4 (pO_2_ 160 mmHg and pCO_2_ 37 mmHg). After the removal of adherent tissues, mainly perivascular fat, CA and MCA segments with a length of 2 mm were mounted on a myograph The contractile responses of the CAs and MCAs were measured in 5 mL PSS maintained at 37 °C and at a pH of 7.4 (pO_2_ 160 mmHg and pCO_2_ 37 mmHg), as previously described [29]. After wall tension normalization, the arteries were stabilized for 40 min. The maximal contractile capacity was triggered in PSS containing 80 mM K^+^ with 3 µM Phe. The endothelial integrity of each artery was controlled after contraction with 2 µM Phe and then 2 µM ACh that induced vasorelaxation. Arteries that did not show endothelium-dependent relaxation (relaxation in response to Ach) were discarded. To characterize the VTD effects, each artery was progressively contracted with U46619 (15 to 90 nM of thromboxane A2 analog by 5 nM increment) to determine the concentration of U46619 that induced 50% of its maximal contraction. PZ (an α1-adrenoceptor inhibitor, 1 µM) or a cocktail of PZ and AP (a muscarinic receptor antagonist, 100 nM) were used to inhibit the effects of NE and ACh released by sympathetic and parasympathetic perivascular nerves, respectively, for 20 min before VTD application. VTD-induced effects were analyzed in the presence of TTX (0.3 and 1 µM) or in a low Na^+^ PSS (in mM: choline chloride, 130; KCl, 3.7; MgSO_4_(7H_2_O), 1.2; NaHCO_3_, 14.9; CaCl_2_(2H_2_O), 1.6; HEPES, 5; KH_2_PO_4_, 1.2; D-glucose, 11). To characterize the VTD-induced vasorelaxation responses observed in the CAs, experiments were performed in the presence or absence of L-NNA (20 µM, a nitric oxide synthase inhibitor), KB-R7943 (a NCX inhibitor; 10 µM), or SEA0400 (a selective NCX1 inhibitor; 1 and 10 µM).

### 5.4. Cell Culture

As a model of ECs, the primary murine mesenteric EC (C57BL/6 mouse primary intestinal mesenteric vascular endothelial cells, M1168, Cell Biologics, Chicago, IL, USA) and Mile Sven 1 MS1 murine pancreatic endothelial cell line (CRL-2279, ATCC^®^, authenticated by ATCC on 26 March 2021) were used. The primary ECs were cultured in complete mouse endothelial cell medium (Cell Biologics) and used from passage 4 until passage 6. The MS1 cells were cultured from passage 8 until passage 20 at 37 °C/5% CO_2_ in DMEM/F12 (PAN-Biotech, Aidenbach, Germany) supplemented with 10% FCS (Eurobio Scientific, Les Ulis, France), 1 mM L-glutamine (PAN-biotech), and 1 mM of penicillin and streptomycin solution (PAN-Biotech).

### 5.5. Quantitative Real-Time Polymerase Chain Reaction

To quantitate NCX transcript expression, total RNAs were extracted from the CAs, isolated mesenteric vascular cells, and MS1 ECs using the RNeasy micro kit (Qiagen, Courtaboeuf, France), as described previously [50]. A total of 300 ng of total RNA was processed for cDNA synthesis using random hexamers and the QuantiTect Reverse Transcription kit (Qiagen). Real-time PCR assays were assessed using a LightCycler 480 Instrument II (Roche, Meylan, France) using the Sybr^®^ Select Master Mix (Applied Biosystems Inc., Lincoln, CA, USA), 6 ng of cDNA in duplicate, and gene-specific primers (see Table 1) previously designed using the Primer3 Software. The cycle threshold method was used to determine differences in transcript expression levels, as described earlier [51]. Amplification specificity was confirmed by one peak-melting curve at the end of the amplification process. To perform the absolute quantification method, 3 cDNA clones of mouse, *Slc8a1-3* (ABIN4099663, ABIN4102124, and ABIN4102129, Genomics, Aachen, Germany) were used. All cDNA sequences were verified and guaranteed by genomics. To calculate the copy numbers of the transcripts, cDNA copies were evaluated using the calibration curve method from the recombinant double-stranded plasmid DNA molecule, as previously described [52]. The cDNA copy numbers of 8 plasmid dilutions were used to establish the calibration curves for each gene, allowing PCR efficiency determination (~100% for each gene). For the relative quantification method, gene expression levels were normalized to the means of the expression levels of 3 validated housekeeping genes using the 2^−ΔCT^ method: *Gapdh* (glyceraldehyde-3-phosphate dehydrogenase), *Hprt* (hypoxanthine phosphoribosyltransferase), and *Gusb* (beta-glucuronidase).

### 5.6. Immunohistochemistry

CAs cryoconserved in an embedding medium OCT compound were transversally cut to 10 µm thick using Cryostat (Leica) at −25 °C and placed on SuperFrostPlus slides (Thermo Fisher Scientific, USA). The artery slices were dried at room temperature. Immunofluorescence staining was performed using the anti-NCX1 mouse primary antibody (1:500, R3F1, Swant AG, Burgdorf, Switzerland). The endothelium was identified with anti-PECAM1 rabbit primary antibodies (anti-CD31 labeled with FITC, 1:50, eBioscience Inc., San Diego, CA, USA). Nuclei were stained with DAPI 10 µg/mL (Molecular probes, Invitrogen, Eugene, OR, USA). All the primary antibodies and DAPI were incubated overnight at 4 °C in a dark room. The artery slices were washed and then incubated with Alexa fluor 568 conjugated goat anti-mouse antibodies (1:200, Invitrogen) for 3 h at 4 °C in dark room. After washing, a drop of aqueous mounting medium (Fluoromount, Sigma-Aldrich Merck, Saint-Louis, MO, USA) was placed on the artery slices, and a coverslip was then put on each slide. After drying for 1 h, fluorescent staining was revealed using a confocal microscope Nikon Eclipse TE2000-S. Imaging acquisition was performed with Metamorph software (Molecular Devices, San Jose, CA, USA). Negative control experiments excluding the primary antibody were carried out to validate the specificity of the immunofluorescence labeling.

### 5.7. Western Blotting

MS1 ECs were lysed in RIPA extraction buffer (50 mM Tris-HCl, 150 mM NaCl, 12 mM Na Deoxycholate, 0.1% SDS, 1% Triton X-100, and protease inhibitor cocktail (Thermo Fisher Scientific)). After homogenization for 30 min at 4 °C, the samples were centrifuged at 14,000× *g* at 8 °C for 20 min. The protein quantity of the supernatant was determined using the Micro BCA Protein Assay Kit (Thermo Fisher Scientific). Either 10 or 30 µg of proteins were separated by SDS-PAGE (10% polyacrylamide gel) and then transferred to a nitrocellulose membrane. The non-specific binding sites were blocked with Tris-buffered saline solution containing 0.1% Tween and 5% BSA for 90 min. Next, the membranes were incubated with rabbit anti-NCX1 (1:500, ANX-011, Alomone Labs, Jerusalem, Israel) or rabbit anti-Pan Na_V_ (1:200, ASC-003, Alomone Labs) primary antibodies overnight at 4 °C. Anti-β-actin (1:10,000, A3854, Sigma-Aldrich Merck) or anti-HSC70 primary antibodies (1:10,000, SC-7298, Santa Cruz Biotechnology, Dallas, TX, USA) were used as loading controls. After washing, the membranes were incubated with the anti-rabbit or -mouse secondary antibodies conjugated to horseradish peroxidase (1:5000, Thermo Fisher Scientific) for 90 min. Proteins were revealed using the Pierce ECL Western Blotting Substrate kit (Thermo Fisher Scientific) and an LAS-3000 imager (Fuji, Tokyo, Japan). Image acquisition was performed with Image Labs (BioRad, Hercules, CA, USA).

### 5.8. Fluorescence Assays

MS1 ECs were plated at a density of 20,000 cells per well (96 wells) in their adequate medium and assayed 24 h after plating using a FlexStation^®^ 3 Benchtop Multi-Mode Microplate Reader (Molecular Devices, Sunnyvale, CA, USA). The cells were washed with Ca^2+^ buffer (Hank’s Balanced Salt Solution, HBSS) and supplemented with 2.5 mM CaCl_2_, 1 mM MgCl_2_, 10 mM HEPES, and 0.5% BSA with a pH adjusted at 7.4. Next, the MS1 ECs were incubated for 1 h at room temperature in the freshly prepared Fura-2-AM buffer (5 µM Fura-2-AM and 0.02% Pluronic^®^-F127 acid dissolved in Ca^2+^ buffer). After washing, they were incubated in Ca^2+^ buffer for 45 min for the complete de-esterification of Fura-2-AM. To evaluate the variation of [Ca^2+^]i, the plates were exposed at 340 nm and 380 nm excitation wavelengths. The fluorescence emission spectra were recorded at 510 nm for 180 s with an acquisition frequency of 0.25 Hz. After a 30 s baseline, ACh (0.1 Nm–10 µM) was automatically injected to stimulate the cells. To evaluate the effects of NCX inhibition, KB-R7943 (10 µM) or SEA0400 (10 µM) were co-injected with ACh. The MS1 ECs were also stimulated with ACh in Na^+^-free buffer (HBSS containing choline chloride instead of Na^+^ and supplemented with 2.5 mM CaCl_2_, 1 mM MgCl_2_, 10 mM HEPES, and 0.5% BSA at a pH of 7.4) or in Ca^2+^-free buffer (HBSS supplemented with 2.5 mM EGTA, 1 mM MgCl_2_, 10 mM HEPES, and 0.5% BSA at a pH of 7.4). To empty the ER Ca^2+^ storage, the cells were treated with the specific inhibitor of SERCA, thapsigargin (5 µM), before ACh-stimulation. Each condition was carried out in 3–5 wells. All experiments were repeated at least three times (*n* = 3–4). Data acquisition was performed with SoftMax Pro 5.4.1 software (Molecular Devices, Sunnyvale, CA, USA).

### 5.9. Statistical Analysis

GraphPad Prism 7.02 (La Jolla, CA, USA) software was used for graph generation and statistical analyses. All data presented are the mean ± SEM. The normality of the data distribution was evaluated using the Shapiro–Wilk test, and significance tests between groups were performed using parametric or non-parametric tests when appropriate. To analyze the significance between two groups, the *t*-test, Wilcoxon test, or Mann–Whitney test were used when appropriate. For multiple comparisons, the Kruskal–Wallis test, followed by Dunn’s post hoc test, was used. Wire myography experiments were repeated with isolated arteries from at least 7 animals. Data from fluorescence assays are the mean ± SEM, calculated from at least 3 independent experiments (*n* = 3–4). The kinetic traces of Fura-2 fluorescence were plotted as an emission ratio (λex 340 nm/λ ex 380nm), and the integration of the fluorescence kinetics (AUC) was used for data analysis. Differences with *p* < 0.05 were considered significant (ns: not significant; * for *p* < 0.05; ** for *p* < 0.01; *** for *p* < 0.001; **** for *p* < 0.0001).

## Figures and Tables

**Table 1:** Sequence of primers used for real-time RT-PCR.

Gene Name	Genbank Accession Number	Protein Name	Forward Primer (5′−3′)	Reverse Primer (5′−3′)
*Scn1a*	NM_018733.2	Na_V_1.1	ttgcaaggggcttctgttta	aggtccacaaactccgtcac
*Scn2a*	NM_001099298.2	Na_V_1.2	gggttgcatatggtttccaa	cccaaggcatttgcagtta
*Scn3a*	NM_018732.3	Na_V_1.3	tcctcagtagtggtgctttgg	gatgtaagtgaagactttgtcagca
*Scn4a*	NM_133199.2	Na_V_1.4	gaaaaccatcacggtcatcc	tccgagagctttttcacagac
*Scn5a*	NM_021544.4	Na_V_1.5	gccagatctctatggcaacc	ttgcccttattcagcacgat
*Scn8a*	NM_001077499.2	Na_V_1.6	ctggtgctggttggacttc	gcccagggcattagctataa
*Scn9a*	NM_001290674.1	Na_V_1.7	gctgagcctatcaatgcaga	acttggcagcatggaaatct
*Scn10a*	NM_001205321.1	Na_V_1.8	tgggtagcttatggcttcaaa	ctatgaggcttgtgagggaga
*Scn11a*	NM_011887.3	Na_V_1.9	ttcataatgtgtggcaactgg	ttattgcacgtggaaccatc
*Scn1b*	NM_011322.3	Na_V_β1	tgctacaagaagattgctgctg	aatggccaggtattctgagg
*Scn2b*	NM_001014761.2	Na_V_β2	caggagtgtaacaattgcacaga	gggttccctgagaactccac
*Scn3b*	NM_178227.4	Na_V_β3	cccctagcttctctagtgctca	ctgtctccgagggtacttctaca
*Scn4b*	NM_001013390.2	Na_V_β4	tgaagaagacccgagagaaga	acccgttctctgtgttgtca
*Chrm1*	NM_001112697.1	M1 receptor	cccccaagatggattgaa	ttttctcagagtaagggcatcac
*Chrm3*	NM_033269.4	M3 receptor	ttcccatcatgatacacacca	aatgtcacgtgcttggtcac
*Chrm5*	XM_006499099.5	M5 receptor	cagcaagctaccctgaatcc	aagcaaaccagactgtgatcatt
*Slc8a1*	NM_001112798.2	NCX1	ccatcctaggcgagcaca	tcgtcttcttaatgagtttgtcca
*Slc8a2*	NM_148946.2	NCX2	cattacgaggatgcttgtgg	catcatccactatcttgacctga
*Slc8a3*	NM_080440.3	NCX3	ggaagccatcactgttagtgc	tgcatgacgtagtcaaagcag
*Nos3*	NM_008713.4	eNOS	ccagtgccctgcttcatc	gcagggcaagttaggatcag
*Pecam1*	NM_008816.3	CD31	ccagtgccctgcttcatc	gcagggcaagttaggatcag
*Gusb*	NM_010368.1	Gus	ctctggtggccttacctgat	cagttgttgtcaccttcacctc
*Hprt*	NM_013556.2	Hprt	aggacctctcgaagtgt	attcaaatccctgaagtactcat
*Gapdh*	NM_008084.2	Gapdh	ccggggctggcattgctctc	ggggtgggtggtccagggtt

## Data Availability

The original contributions presented in this study are included in the article/Supplementary Materials. Further inquiries can be directed to the corresponding author(s).

## Supplementary Materials

The following supporting information can be downloaded at https://www.mdpi.com/article/10.3390/toxins16120533/s1. Figure S1: Effects of SN-6 on ACh-induced Ca^2+^ response in MS1 ECs, Figure S2: Effects of veratridine on first order mesenteric arteries from mice.

## Abbreviations

The following abbreviations are used in this manuscript:

ACh	acetylcholine
AP	atropine
AUC	area under curve
CA	cecocolic artery
[Ca^2+^]i	intracellular Ca^2+^ concentration
EC	endothelial cell
ER	endoplasmic reticulum
FOMA	first-order mesenteric artery
IP3	inositol 1,4,5 triphosphate
L-NNA	N(ω)-nitro-L-arginine
MA	mesenteric artery
MCA	middle cecolic artery
MS1	Mile Sven 1 murine
M3-mAChR	muscarinic receptor subtype 3
[Na^+^]i	intracellular Na^+^ concentration
Na_V_ channel	voltage-gated Na^+^ channel
NE	norepinephrine
Phe	phenylephrine
PZ	prazosin
TTX	tetrodotoxin
TTX-R	resistant to tetrodotoxin
TTX-S	sensitive to tetrodotoxin
VSMC	vascular smooth muscle cell
VTD	veratridine
