# Supplementary material for: Veratridine Induces Vasorelaxation in Mouse Cecocolic Mesenteric Arteries
Source: Toxins (Basel). 2024 Dec 10;16(12):533. doi: 10.3390/toxins16120533 (PMC11679225; doi:10.3390/toxins16120533)
Supplement: Supplementary file 1 [file toxins-16-00533-s001.zip › toxins-3317695-supplementary.pdf]

Supplementary Materials: Veratridine Induces Vasorelaxation in Mouse Cecocolic Mesenteric Arteries

Joohee Park, Christina Sahyoun, Jacinthe Frangieh, Léa Réthoré, Coralyne Proux, Linda Grimaud, Emilie Vessières, Jennifer Bourreau, César Mattei, Daniel Henrion, Céline Marionneau, Ziad Fajloun, Claire Legendre and Christian Legros

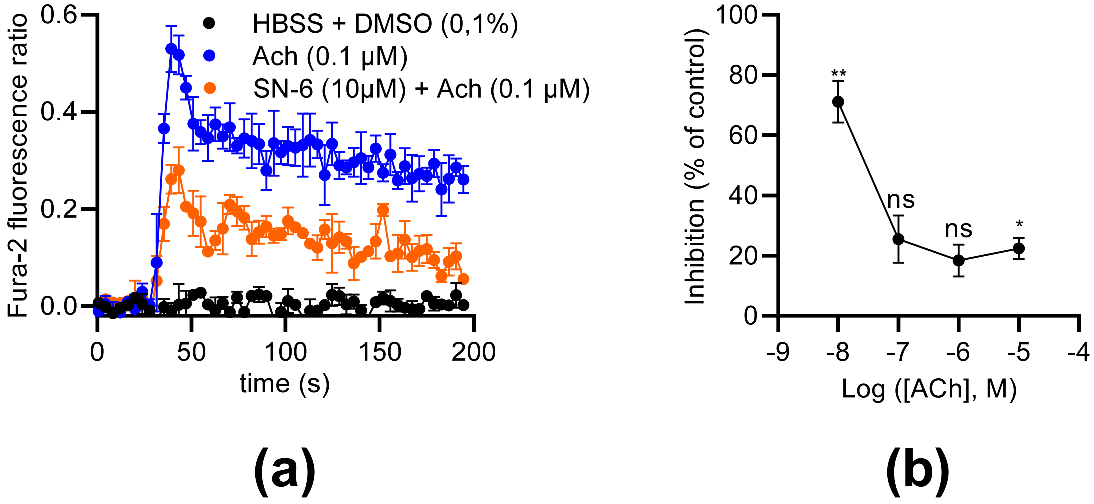

Figure S1. Effects of SN-6 on ACh-induced Ca^{2+} response in MS1 EC. **(a)** Example of kinetic traces of Fura-2 fluorescence emission ratio, illustrating the effects of ACh (0.1 µM) injected alone or with SN-6 (10 µM) at 30 s. As a negative control, DMSO (1%) in HBSS, corresponding to the vehicle was injected. **(b)** The graph illustrates the inhibition (% of control) induced by SN-6, as a function of ACh concentration. Data are mean ± SEM of three independent experiments. Statistical significances were determined using the Wilcoxon test (**b**) (* $p < 0.05$; ** $p < 0.01$).

FURA-2 Fluorescence Assays

MS1 EC were plated at a density of 20,000 cells per wells (96 wells) in their adequate medium and assayed 24 hours after plating using a FlexStation® 3 Benchtop Multi-Mode Microplate Reader (Molecular Devices, Sunnyvale, CA, USA). The cells were washed with Ca^{2+} buffer (Hank's Balanced Salt Solution, HBSS), supplemented with 2.5 mM $CaCl_2$, 1 mM $MgCl_2$, 10 mM HEPES and 0.5% BSA with pH adjusted at 7.4. Next, MS1 EC were incubated for 1 h at room temperature in the freshly prepared Fura-2-AM buffer (5 µM Fura-2-AM and 0.02% Pluronic®-F127 acid dissolved in Ca^{2+} buffer). After washing, they were incubated in Ca^{2+} buffer during 45 min for a complete de-esterification of Fura-2-AM. To evaluate the variation of $[Ca^{2+}]i$, the plates were exposed at 340 nm and 380 nm excitation wavelengths. The fluorescence emission spectra were recorded at 510 nm for 180 s with 0.25 Hz of acquisition frequency. After 30 s baseline, ACh (0.1 nM-10 µM) was automatically injected to stimulate the cells. To evaluate the effects of NCX inhibition, SN-6 (10 µM) was co-injected with ACh. Each condition was carried out in 3-5 wells. All experiments were repeated at least three times (n = 3–4). Data acquisition was performed with SoftMax Pro 5.4.1 software (Molecular Devices, Sunnyvale, CA, USA).

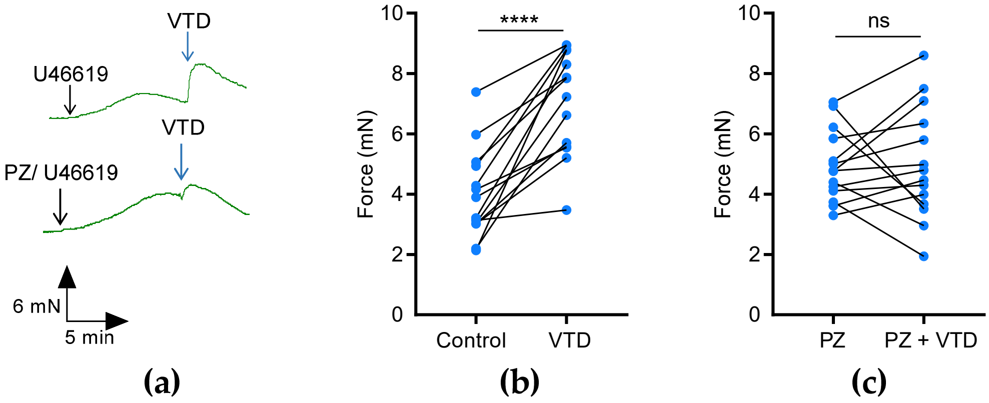

Figure S2. Effects of veratridine on first order mesenteric arteries from mice. **(a)** Representative myograph traces illustrating contraction of first order mesenteric arteries (FOMA) from mice triggered by veratridine (VTD, 30 μM) in the absence (upper trace) and in the presence (lower trace) of 1 μM of prazosin (PZ). **(b,c)** Graphs showing the connected scatter plot of individual values of wall tension levels (Force, in mN) for each artery, before and after application of VTD in the absence **(b)** or the presence **(c)** of PZ (1 μM). FOMA were isolated from male (n=7) and female (n=7) mice. Significance between groups was determined with paired t test (****: p<0.001; ns: non-significant, p=0.8971).
